# Chronic dialysis, *NAT2* polymorphisms, and the risk of isoniazid-induced encephalopathy – case report and literature review

**DOI:** 10.1186/s12882-017-0703-6

**Published:** 2017-09-04

**Authors:** Stefan Matei Constantinescu, Benoit Buysschaert, Vincent Haufroid, Franck Broly, Michel Jadoul, Johann Morelle

**Affiliations:** 1 Division of Nephrology, Cliniques universitaires Saint-Luc, Avenue Hippocrate 10, B-1200 Brussels, Belgium; 2 Department of Clinical Chemistry, Cliniques universitaires Saint-Luc, Brussels, Belgium; 3 Institut de Recherche Expérimentale et Clinique, Université catholique de Louvain, Brussels, Belgium; 4 Department of Toxicology and Genopathy, Biology Pathology Center, Lille University Hospital, Lille, France; 5 University of Lille 2, Lille, France

**Keywords:** *Mycobacterium tuberculosis*, End-stage renal disease, Hemodialysis, Slow acetylator, Encephalopathy, Case report

## Abstract

**Background:**

Isoniazid is the most widely used anti-tuberculosis agent, yet it may lead to life-threatening complications.

**Case presentation:**

Here we report the case of a chronic hemodialysis patient who developed severe encephalopathy after the start of isoniazid. Blood levels of isoniazid were elevated, and acetyl-isoniazid over isoniazid ratio was decreased 3 h after intake of the medication, suggesting that a slow acetylator phenotype may have contributed to drug toxicity, in addition to pyridoxal phosphate removal by dialysis. This hypothesis was confirmed by sequencing of *NAT2*, the gene responsible for isoniazid elimination, and identification of *NAT2* polymorphisms compatible with a slow acetylator phenotype. Isoniazid withdrawal along with supplementation using high doses of pyridoxine successfully reversed the drug toxicity. Isoniazid toxicity occurs in populations at risk, including patients with chronic kidney failure or *NAT2* polymorphisms, who have a disturbed metabolism of pyridoxine or isoniazid, respectively, and those on renal replacement therapies, in whom pyridoxal phosphate – the active metabolite of pyridoxine – is inadvertently removed by dialysis.

**Conclusions:**

Physicians should be aware of the increased risk of isoniazid toxicity in patients on dialysis and in those with a slow acetylator phenotype conferred by *NAT2* polymorphisms. Adaptation of prescription – either with higher doses of pyridoxine or decreased doses of isoniazid, respectively – has been suggested to reduce the risk of potentially life-threatening toxicity of isoniazid.

## Background

Despite recent advances and the fact that nearly all cases can be cured, tuberculosis remains one of the world’s biggest threats. In 2014, 9.6 million people developed tuberculosis and 1.5 million died from the disease [1]. Isoniazid is one of the oldest but also the most widely used anti-tuberculosis agent; it is easily administered, inexpensive, and exerts a high bactericidal activity against *Mycobacterium tuberculosis*.

However, isoniazid may lead to serious adverse events, including hepatotoxicity, peripheral neuropathy and encephalopathy, especially in populations at risk [2]. The following case illustrates the impact of end-stage renal disease and *NAT2* polymorphisms on the risk of isoniazid toxicity, and discusses how these life-threatening complications may be prevented and managed.

## Case presentation

A 71-year-old African woman on chronic dialysis was referred to our institution because of unexplained lethargy and altered state of consciousness. Hemodialysis – using high-efficiency/high-flux membranes - had been initiated 5 years earlier because of end-stage nephrosclerosis. No significant complication occurred since the onset of dialysis. Her neurological status was normal until 6 weeks before her transfer, when she developed confusion and, over a few days, unresponsiveness. An extensive workup had been performed at another university hospital and found no evidence for a metabolic or vascular etiology, nor meningo-encephalitis; the electroencephalogram showed severe generalized abnormalities in keeping with a diagnosis of encephalopathy. The patient was transferred to our hospital for further investigation, upon request of the family.

**Fig. 1:**
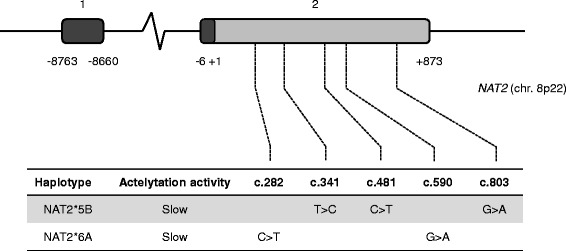
Structure of the *NAT2* gene and polymorphisms identified in our patient. The relative position of Exon 1, as well as the first (+1) and last (+873) positions of the open reading frame of Exon 2 are indicated. Vertical dotted lines show the positions of the single nucleotide polymorphisms identified in our patient. Haplotypes arising from the combinations of SNPs are shown in the table, with their associated acetylation activity

At admission, blood pressure was 120/70 mmHg and heart rate regular at 86 bpm. She was somnolent, opened her eyes to verbal stimuli, uttered only sounds and had an avoiding response to pain. She scored 9/15 on the Glasgow coma scale. Two months before her transfer, she had undergone a workup for a chronic dry cough, including chest computed tomography-scan, positron emission tomography, bronchoalveolar lavage and interferon-gamma release assay. The results suggested latent tuberculosis infection, and the patient was prescribed isoniazid 300 mg o.d. along with weekly pyridoxine 250 mg. Altered mental status with somnolence then unresponsiveness developed two weeks after the start of isoniazid. Three hours after intake of isoniazid, blood level of the drug was 2.89 mg/l (normal range 1–2 mg/l), and the ratio of inactive acetyl-isoniazid to isoniazid was 0.33, a result compatible with a slow acetylator phenotype (<0.48, slow acetylators; >0.77, fast acetylators) [3]. Slow acetylator phenotype was confirmed by sequencing of *NAT2* – the gene encoding the human isoenzyme arylamine N-acetyltransferase 2 (NAT2) (NM_000015.2) – which showed a heterozygous status for the variants c.282C > T(rs1041983), c.341 T > C(rs1801280), c.481C > T(rs1799929), c.590G > A(rs1799930) and c.803G > A(rs1208), compatible with a NAT2*5B/*6A genotype (Fig. 1).

Isoniazid was discontinued soon after admission in our unit, and the patient was given high doses of oral pyridoxine (250 mg daily, equivalent to 1750 mg weekly). A remarkable improvement of the patient’s condition ensued, and Glasgow coma scale was 15/15 ten days later. She was discharged two weeks after admission and was prescribed physical therapy at home, in addition to continuing maintenance hemodialysis. Three months after discharge she had returned to her previous condition, being again able to have a full conversation and walk on her own. The life-threatening complication of isoniazid therapy, along with the debatable indication of treatment, prompted watchful waiting rather than reintroduction of any anti-tubercular treatment. Clinical follow-up was excellent, and a computed tomography of the chest performed one year later did not show any sign suggestive of tuberculosis infection.

## Discussion

The present case illustrates the risk of potentially life-threatening toxic side effects of isoniazid in patients at risk, including end-stage renal disease patients on dialysis and/or those harboring a slow acetylator phenotype conferred by *NAT2* polymorphisms.

More than 2 million people worldwide have end-stage renal disease and require dialysis as a life-saving therapy [4]. In the dialysis population, *Mycobacterium tuberculosis* infection has a high prevalence, ranging between 5 and 25%, and isoniazid is frequently used to prevent reactivation [5]. Latent tuberculosis is frequently diagnosed in renal transplant candidates, who all should be screened during pre-transplantation evaluation, as the risk of active tuberculosis in transplant recipients is estimated to be 20–74 times higher than in the general population [6]. Patients on dialysis are at risk for isoniazid toxicity, because of altered pyridoxine metabolism resulting in severe deficiency in pyridoxal phosphate (the active form of pyridoxine), and extensive removal of pyridoxal phosphate (a small molecule of 247 Da) by renal replacement therapies [7]. While the recommended dose of pyridoxine supplementation in case of isoniazid treatment is usually 10–25 mg per day, [8] it needs to be increased to >100 mg/day to prevent drug toxicity among patients on dialysis [7]. Patients on high-flux high-efficiency hemodialysis – as our patient – are particularly at risk of pyridoxal phosphate depletion because of higher clearances during dialysis sessions [9].

Isoniazid is primarily metabolized by the polymorphic arylamine NAT2. NAT2 activity is genetically determined and basically relies on the number of active alleles in *NAT2* gene (NAT2*4 and *12), thereby providing a molecular mechanism for the large inter-individual variability in toxicity and efficacy of isoniazid [10]. Individuals are therefore classified as rapid metabolizers if they have one or more NAT2*4 alleles, and slow metabolizers only if they carry two slow metabolizer variants, as our patient (Fig. 1). During treatment with the standard regimen, slow acetylators carry a higher risk of drug toxicity, while rapid acetylators are prone to treatment failure, due to insufficient exposure to isoniazid. As a result, it has been suggested that *NAT2* genotype may help determining the dose of isoniazid in individual patients [11]. The appropriateness of such a pharmacogenetics-based has been recently validated in a large randomized controlled trial. *NAT2* genotype-guided regimen significantly reduced the risk of both toxicity and treatment failure, in slow and rapid acetylators, respectively [12]. In our patient, the combination of increased isoniazid and decreased acetyl-ionazid levels pointed towards a slow acetylator phenotype, an hypothesis that was further verified by the identification of NAT2*5B/*6A genotype. Intriguingly, Sub-Saharan populations show a high prevalence (>70%) of slow acetylator phenotype related to *NAT2* polymorphisms, possibly as a consequence of selective pressure related to the chemical environment, climate, biome, and dietary habits [13].

## Conclusions

In conclusion, the combination of end-stage renal disease, removal of pyridoxal phosphate by hemodialysis, and deleterious *NAT2* polymorphisms, contributed to the severe toxicity of isoniazid in our patient. Drug withdrawal and administration of high doses of pyridoxine rapidly reversed drug toxicity. Physicians should be aware of the increased risk of isoniazid toxicity in patients on dialysis and in those with a slow acetylator phenotype conferred by *NAT2* polymorphisms. Adaptation of prescription – either with higher doses of pyridoxine or decreased doses of isoniazid, respectively – has been suggested to effectively reduce the risk of potentially life-threatening toxicity of isoniazid, while avoiding treatment failure. Future studies will need to validate *NAT2* genotype-guided drug dosage of isoniazid as a safe and appropriate alternative to standard dose regimen.

## Abbreviation

NAT-2: N-acetyltransferase 2

## References

[1] World Health Organization. Global Tuberculosis Report 20^th^ Edition; 2015.

[2] Adams P, White C (1965). Isoniazid-induced encephalopathy. Lancet.

[3] Miscoria G, Leneveu A, Walle C (1988). Application of a method of analysis using high performance liquid chromatography of isoniazid and acetylisoniazid to determine the phenotype of acetylation. Ann Biol Clin (Paris).

[4] Liyanage T, Ninomiya T, Jha V (2015). Worldwide access to treatment for end-stage kidney disease: a systematic review. Lancet.

[5] Korzets A, Gafter U (1999). Tuberculosis prophylaxis for the chronically dialysed patient - yes or no?. Nephrol Dial Transplant.

[6] Horne DJ, Narita M, Spitters CL, Parimi S, Dodson S, Limaye AP (2013). Challenging issues in tuberculosis in solid organ transplantation. Clin Infect Dis.

[7] Siskind MS, Thienemann D, Kirlin L (1993). Isoniazid-induced neurotoxicity in chronic dialysis patients: report of three cases and a review of the literature. Nephron.

[8] CDC 24/7: Saving Lives, Protecting People. Atlanta: Centers for Disease Control and Prevention. Latent Tuberculosis Infection: A Guide for Primary Health Care Providers. Updated 2013 April 3 Available from: http://www.cdc.gov/tb/publications/ltbi/treatment.htm.

[9] Kasama R, Koch T, Canals-Navas C, Pitone JM (1996). Vitamin B6 and hemodialysis: the impact of high-flux/high-efficiency dialysis and review of the literature. Am J Kidney Dis.

[10] Mashimo M, Suzuki T, Abe M (1992). Molecular genotyping of N-acetylation polymorphism to predict phenotype. Hum Genet.

[11] Kinzig-Schippers M, Tomalik-Scharte D, Jetter A (2005). Should we use N-acetyltransferase type 2 genotyping to personalize isoniazid doses?. Antimicrob Agents Chemother.

[12] Azuma J, Ohno M, Kubota R (2013). NAT2 genotype guided regimen reduces isoniazid-induced liver injury and early treatment failure in the 6-month four-drug standard treatment of tuberculosis: a randomized controlled trial for pharmacogenetics-based therapy. Eur J Clin Pharmacol.

[13] Podgorná E, Diallo I, Vangenot C (2015). Variation in NAT2 acetylation phenotypes is associated with differences in food-producing subsistence modes and ecoregions in Africa. BMC Evol Biol.

